# Comparative Single-Cell Genomics of Chloroflexi from the Okinawa Trough Deep-Subsurface Biosphere

**DOI:** 10.1128/AEM.00624-16

**Published:** 2016-05-02

**Authors:** Heather Fullerton, Craig L. Moyer

**Affiliations:** Department of Biology, Western Washington University, Bellingham, Washington, USA; University of Tennessee and Oak Ridge National Laboratory

## Abstract

Chloroflexi small-subunit (SSU) rRNA gene sequences are frequently recovered from subseafloor environments, but the metabolic potential of the phylum is poorly understood. The phylum Chloroflexi is represented by isolates with diverse metabolic strategies, including anoxic phototrophy, fermentation, and reductive dehalogenation; therefore, function cannot be attributed to these organisms based solely on phylogeny. Single-cell genomics can provide metabolic insights into uncultured organisms, like the deep-subsurface Chloroflexi. Nine SSU rRNA gene sequences were identified from single-cell sorts of whole-round core material collected from the Okinawa Trough at Iheya North hydrothermal field as part of Integrated Ocean Drilling Program (IODP) expedition 331 (Deep Hot Biosphere). Previous studies of subsurface Chloroflexi single amplified genomes (SAGs) suggested heterotrophic or lithotrophic metabolisms and provided no evidence for growth by reductive dehalogenation. Our nine Chloroflexi SAGs (seven of which are from the order Anaerolineales) indicate that, in addition to genes for the Wood-Ljungdahl pathway, exogenous carbon sources can be actively transported into cells. At least one subunit for pyruvate ferredoxin oxidoreductase was found in four of the Chloroflexi SAGs. This protein can provide a link between the Wood-Ljungdahl pathway and other carbon anabolic pathways. Finally, one of the seven Anaerolineales SAGs contains a distinct reductive dehalogenase homologous (*rdhA*) gene.

**IMPORTANCE** Through the use of single amplified genomes (SAGs), we have extended the metabolic potential of an understudied group of subsurface microbes, the Chloroflexi. These microbes are frequently detected in the subsurface biosphere, though their metabolic capabilities have remained elusive. In contrast to previously examined Chloroflexi SAGs, our genomes (several are from the order Anaerolineales) were recovered from a hydrothermally driven system and therefore provide a unique window into the metabolic potential of this type of habitat. In addition, a reductive dehalogenase gene (*rdhA*) has been directly linked to marine subsurface Chloroflexi, suggesting that reductive dehalogenation is not limited to the class Dehalococcoidia. This discovery expands the nutrient-cycling and metabolic potential present within the deep subsurface and provides functional gene information relating to this enigmatic group.

## INTRODUCTION

The phylum Chloroflexi, colloquially known as the green nonsulfur bacteria, has been recognized as both ubiquitous and diverse using small-subunit (SSU) rRNA in molecular studies (1). Environmental molecular microbial surveys have shown Chloroflexi to be abundant in marine, intertidal, and freshwater surface and subsurface sediments (2); however, the phylum has only a few cultured representatives (3). The cultured representatives of the group have a wide range of metabolic activities, including fermentation, anoxygenic photosynthesis, nitrite oxidation, and reductive dehalogenation (3–7). Due to the metabolic diversity across the phylum, metabolic activity cannot be inferred based solely on phylogeny. (Select cultured representatives are listed in Table S1 in the supplemental material, along with their metabolic classifications.)

In comparisons among Chloroflexi-containing sites that were rich in methane hydrates and organic carbon off the Peru and Cascade Margins, Chloroflexi SSU rRNA gene sequences were more numerous at the organic-rich sites (8). A metagenomic study of the Peru Margin showed Chloroflexi to be present at a broad range of depths and to compose 12 to 16% of the total genes identified (9). In addition to the Peru Margin, Caldilineae- and Anaerolineae-related sequences comprised a major portion of the subsurface bacterial community within a forearc basin off Sumatra and the Black Sea (10). Unlike their photosynthesizing or terrestrial relatives, none of these deep-sea strains are yet in culture, so virtually nothing is known about their metabolic potential (11). Chloroflexi have been previously identified in a subsurface hydrothermal habitat at Iheya North, Mid-Okinawa Trough (12). It is likely that similar subsurface Chloroflexi have the potential for growth, since Chloroflexi sequences were recovered from an *in situ* colonization experiment within a borehole, indicating recent growth on previously uninhabited surfaces (13).

With the advances in single-cell genomics, it is no longer required to have axenic bacterial cultures to obtain an individual genome, and in some cases, recovered single amplified genomes (SAGs) have been reported to be more than 90% complete (14, 15). The SAG approach can reveal the metabolic functions of the abundant and frequently detected Chloroflexi for comparison to those of the sequenced photosynthetic or terrestrial isolates. Previously studied Chloroflexi SAGs belong to the class Dehalococcoidia and contain genes for autotrophic growth via the Wood-Ljungdahl pathway, also known as the reductive acetyl coenzyme A (CoA) pathway (16, 17). Dehalococcoidia SAGs from the Peru Trench, Dsc1 and DscP2, were recovered from 7.3 m below the seafloor (bsf) at a seawater depth of 5,086 m from Integrated Ocean Drilling Program (IODP) site 1230 and a single SAG from a depth of 16.3 m in Aarhus Bay, SCGC AB-539-J10 (abbreviated DEH-J10), was recovered at just 0.16 m bsf. Therefore, Dsc1 and DscP2 are the only deep-subsurface Chloroflexi that have been analyzed for their metabolic potential prior to this study. Previously, *rdhA* genes have been identified from isolates within the order Dehalococcoidales of the phylum Chloroflexi (18) or from dehalogenating enrichment cultures (19). Furthermore, isolated strains of Dehalococcoides mccartyi depend on dehalogenation as their sole means of respiration via the RdhA proteins (18). No *rdhA* genes were identified in DscP2, Dsc1, or DEH-J10, and it was unclear if these organisms had heterotrophic or autotrophic metabolisms (16, 17). In marine subsurface sediments, *rdhA* homologues have been detected by PCR and in metagenomic studies from around the Pacific Ocean, and dehalogenation activity has been shown in sediment microcosms, suggesting organohalide respiration is an important energy-yielding pathway in subseafloor microbial ecosystems (20, 21).

Hydrothermal vents circulate reduced chemicals and seawater, providing microbial niches that can support a larger biomass than the surrounding ocean floor (22), making vent fields some of the most biologically active regions in the deep sea (23). These systems harbor diverse microbial communities in and around vent orifices and within surrounding subsurface environments. The Okinawa Trough contains both hemipelagic and volcanic sediments, sometimes over 1 km thick, providing hydrothermal systems with abundant H_2_, CO_2_, CH_4_, NH_4_, H_2_S, and CO derived from sedimentary organic matter and from magmatic gases. The Iheya North hydrothermal field is an area of diffuse flow surrounding a focused-flow high-temperature vent system, which makes it a promising environment to study functionally active and metabolically diverse microbes (24, 25). The abundant energy and carbon supplied through hydrothermal-vent circulation provide an ideal location to study elusive microbes, such as the Chloroflexi, within the subsurface biosphere.

**FIG 1:**
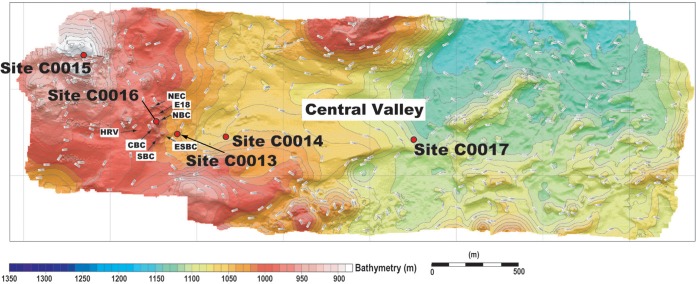
Detailed bathymetry of the Iheya North hydrothermal field and the central valley at Iheya North Knoll, with the location of sites C0013 through C0017, drilled during IODP expedition 331. HRV, High Radioactive Vent mound; CBC, Central Big Chimney; SBC, South Big Chimney mound; NEC, North Edge Chimney mound; E18, Event Marker 18 mound; NBC, North Big Chimney mound; ESBC, “Ese” South Big Chimney mound. (Map modified from reference 25 and reprinted from reference 24.)

Five locations were chosen for drilling at Iheya North hydrothermal field (Fig. 1) as part of IODP expedition 331 (Deep Hot Biosphere). Based on the initial heat flow results, a hydrodynamic model of fluid flow throughout the Iheya North hydrothermal field was documented, where variation in chemical and physical processes, including the formation of both brine- and vapor-rich hydrothermal fluids (i.e., phase separation and phase partitioning), was likely generated by underlying high temperatures coupled with a complex hydrogeologic structure. The recovered drill cores were described as interbedded hemipelagic muds with strongly pumiceous volcanoclastic sediments; however, there was minimal hydrothermal alteration in the cooler zones, generally within the upper 30 m bsf, showing evidence of microbial activity (24, 25). The cooler sites chosen for this study were (i) site C0015, located ∼600 m northwest of the active vents on the crest of a hill, which represented a potential diffuse (i.e., high seawater mixing) outflow migration path for hydrothermal fluids, and (ii) site C0017, located ∼1,550 m to the east of the active vents, representing an area of hydrothermal recharge or an inflow path for ambient seawater (24, 25).

We examined and compared nine SAGs belonging to the phylum Chloroflexi recovered from the Iheya North hydrothermal field in the Mid-Okinawa Trough during IODP expedition 331 (24, 25). In contrast to previously studied Chloroflexi SAGs, an *rdhA* gene was identified in an Anaerolineales representative. This study expands the known metabolic repertoire of this elusive group of microbes and provides insights into their metabolic potential.

## MATERIALS AND METHODS

### Sample collection.

Subsurface sediments were collected on IODP expedition 331 (Deep Hot Biosphere) from 1 September through 4 October 2010 (Fig. 1). Onboard contamination testing of sites C0015 (126°53′E, 27°47′N; hole B; section 1H-5; 5.6 m bsf) and C0017 (126°55′E, 27°47′N; hole C; section 1H-7; 26.6 m bsf) found no indication of interior-core contamination using fluorescent microspheres (both holes C0015B and C0017C) and perfluorocarbon tracer (hole C0017C only). The sample from hole C0017C was also verified by PCR-generated phylotype comparisons based on 97% similarity to phylotypes obtained from drilling mud at a contamination level of 1% or less (26). Subsamples were aseptically collected from the interiors of whole-round cores and stored in cryovials with 27% (vol/vol) glycerol at −80°C.

### Single-cell source.

Core depths were chosen from sites C0015 and C0017, which were characterized as weakly oxidized pumiceous gravels with no detected sulfide mineralization and less than 0.1 wt% total organic carbon, total nitrogen, and total sulfur (25). The selected samples for single-cell genomes were from subsurface depths of 5.6 m bsf from hole C0015B and from 26.6 m bsf from hole C0017C. Temperatures were estimated at ∼10.5°C for C0015B and ∼8.1°C for C0017C at these depths. Details of geochemistry and lithography have been previously described (12, 24, 25).

### Single-cell sorting, amplification, sequencing, and annotation.

Samples from sites C0015 and C0017 (Fig. 1) were diluted with 1 ml of filter-sterilized artificial seawater (27), making a slurry, and then passed through a 90-μm nylon mesh filter twice and centrifuged at ∼500 × *g* for 2 min to produce a particle-free cell suspension. The suspension was then processed using fluorescence-activated single-cell sorting at the Single Cell Genome Center (SCGC) at Bigelow Laboratory for Ocean Sciences. Single-cell sorting and multiple displacement amplification (MDA) have been previously described (28). The amplified SSU rRNA gene sequences (27F/907R) were classified using the Ribosomal Database Project (RDP) online classifier (28, 29). Based on their SSU rRNA gene identities, nine Chloroflexi SAGs (of the total 29 unique MDA reactions identified after cell sorting) were chosen for whole-genome sequencing. These SAGs were sequenced and assembled, and contamination was checked by the SCGC, using previously well-described parameters (28, 29). Assembly was done using SPAdes v.3.0.0 (30). All contigs were compared to ensure no cross-contamination among SAGs and the NCBI nt database, which was followed by tetramer principal-component analysis as previously described (31–33). These analyses revealed no contamination. The full name for each of the SAGs was shortened, e.g., Anaerolineales bacterium SCGC AC-711-B22 was shortened to An-B22. Phylogeny was abbreviated as follows: Anaerolineales to An, Dehalococcoidales to De, and Thermoflexales to Th. The assembled genomes were annotated using RAST (34). Gene annotations were compared to NCBI GenBank via BLASTn, and the results can be found in Tables S2 to S4 in the supplemental material.

The Anaerolineales SAGs were compared to the genome of Anaerolinea thermophila UNI-1 (GenBank accession number NC_014960) and the single Thermoflexales SAG to that of Thermoflexus hugenholtzii JAD2 (NCBI BioProject PRJNA195829), as they were determined to be their closest respective relatives. The type strain A. thermophila UNI-1 was isolated from an anaerobic granular sludge reactor treating fried soybean curd manufacturing wastewater in Japan (35), while the type strain T. hugenholtzii JAD2 was isolated from the sediment of Great Boiling Spring in Nevada (36). Both are considered thermophilic, Gram-negative, non-spore-forming, heterotrophic bacteria that grow in multicellular filaments (36, 37).

### Phylogenetic analysis.

SSU rRNA gene sequences and phylogenetic relatives were aligned using the Silva SINA aligner (38). For the *rdhA* analysis, amino acids were aligned using ClustalW within Geneious (39, 40). The resulting alignments were manually screened and then used to create a phylogenetic consensus tree using MrBayes within Geneious (41). Parameters included using the HKY85 substitution model, the chain length set at 1,100,000, and a subsampling frequency of 200. Priors were set with an unconstrained branch length. The average nucleotide identity (ANI) was calculated for the SAGs and selected genomes, with the BLAST parameters as previously described (42).

### Genome completeness estimates.

Genome completeness estimates were determined with BLASTP using predicted amino acid sequences against a set of single-copy core genes (43). To be considered valid, all proteins must have at least 30% identity over at least 30% of the length of the core gene (44). The core gene group is made up of 66 previously established genes belonging to a nonredundant list as examined by gene ontology (GO) annotations (44, 45).

### Accession numbers.

The SSU rRNA gene sequences obtained from MDA have been submitted to the NCBI GenBank database (accession numbers KT119838 to KT119846). All the SAGs have been made public in the Integrated Microbial Genomes (IMG) database (IMG submission identifiers [IDs] 68650, 69642 to 69645, 69647 to 69649, and 69684).

## RESULTS AND DISCUSSION

### Single-cell sorts.

**FIG 2:**
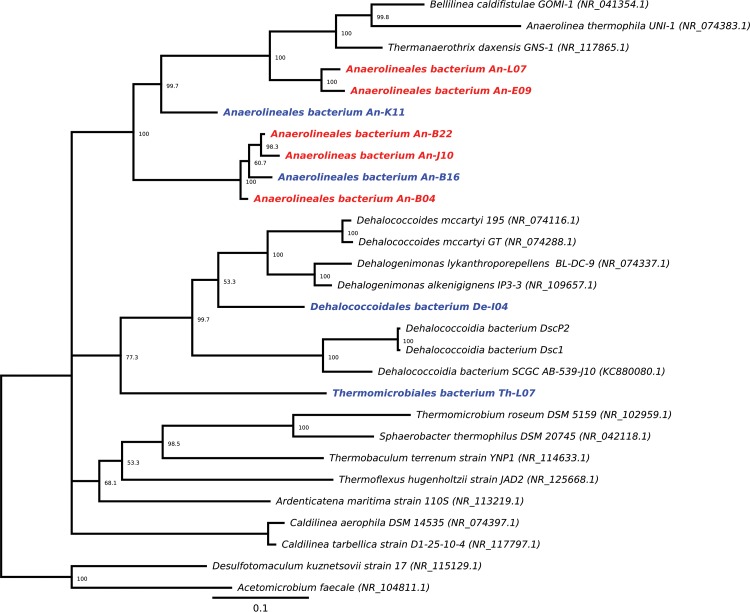
Unrooted Bayesian tree of Chloroflexi SSU rRNA gene sequences from the Okinawa Trough. The samples are color coded for the collection site, with C0015 and C0017 in red and blue, respectively. The numbers at the nodes represent percent consensus support. The scale bar represents 1 nucleotide substitution per 10 positions.

Classification of SSU rRNA gene sequences by taxonomic analysis showed Chloroflexi to be the most abundant within the two subsurface habitats with respect to the total number of single cells recovered. Nine Chloroflexi SSU rRNA gene sequences were recovered, four from hole C0015B and five from hole C0017C. Of the nine Chloroflexi SAGs detected, seven belonged to the order Anaerolineales, with one each belonging to the orders Dehalococcoidales and Thermoflexales (Table 1). Phylogenetic placement was not correlated with the location from which the SAG was recovered (Fig. 2). A previous study from IODP expedition 331 examined cloned SSU rRNA gene sequences from site C0017 for bacterial taxonomic classification and showed that Chloroflexi were one of the main constituents of the microbial communities from 0.7 to 74.9 m bsf, crossing multiple sediment types, including oxygenated and anoxic layers, as well as the gradients among these layers, showing the phylum to be ubiquitous (12).

**TABLE 1:** Assembly statistics and phylogenetic classification as determined by SSU rRNA gene sequences from Chloroflexi SAGs

SAG name	Order	Collection site	Assembled length (bases)	Estimated genome recovery (%)	Max contig length (bases)	Contig count	GC content (%)	No. of predicted genes	No. of RNAs	No. of tRNAs
An-E09	Anaerolineales	C0015	153,247	1.5	50,084	11	55.6	175	2	2
De-I04	Dehalococcoidales	C0017	187,486	4.5	30,599	21	47.8	211	5	5
Th-L07	Thermoflexales	C0017	462,464	10.6	46,198	46	57.63	447	10	7
An-K11	Anaerolineales	C0017	493,899	18.2	46,925	50	56.5	455	18	18
An-B04	Anaerolineales	C0017	615,816	16.7	66,121	63	56.6	588	5	4
An-L07	Anaerolineales	C0015	800,722	16.7	125,785	72	58.4	818	11	11
An-J10	Anaerolineales	C0015	1,010,824	19.7	84,943	62	56.9	967	21	20
An-B16	Anaerolineales	C0017	1,258,475	31.8	101,499	82	56.9	1,214	35	33
An-B22	Anaerolineales	C0015	1,423,460	62.1	67,055	134	56.8	1,411	33	26

### Genome composition.

Overall, 6.4 Mb of Chloroflexi genomic data has been analyzed. The assembled sequence length was variable from 0.15 Mb to 1.4 Mb, representing 1.5% to 62.1%, with an average of 20.2%, completeness based on core GO annotations (Table 1). tRNA recovery estimates revealed a slightly higher percent recovery (4.0% to 66%, with an average of 28.3%) overall with an estimated average genome size of 3.15 Mb (see Table S5 in the supplemental material). While some of the recovered SAGs were small, all the annotated genes could be conclusively linked to a phylogenetic group based on the classifications originating from the single-cell sorts.

The seven Anaerolineales SAGs and the Thermoflexales SAG had similar GC contents at approximately 55%. The remaining SAG belongs to the order Dehalococcoidales and had a GC content of 47%. Comparison of the SSU rRNA gene sequences across the nine Chloroflexi SAGs showed that two SAGs, An-B22 and An-J10, had nearly identical sequences. Whole-genome comparisons between An-B22 and An-J10 showed an ANI of 98% (42). Both of the SAGs were collected from site C0015 and belong to the order Anaerolineales. In addition, Th-L07 had an ANI of 98% to An-B22 (see Fig. S1 in the supplemental material). By SSU rRNA gene classification, strain Th-L07 is classified as a member of the Thermoflexales and has 85% identity to An-B22.

Besides An-B22 having high identity to An-J10 and Th-L07, no other comparison of SAGs yielded an ANI greater than 91%. SAG Th-L07 is only 10% complete and is roughly one-third the size of An-B22. The pairwise ANI values take into account only the genes present in the smaller of the genomes compared, and though a conservative estimate, this can be misleading across variable-size genomes. It has been suggested that only genomes that are at least 20% complete provide accurate species ANI values (46); however, ANIs have been shown to differentiate among operational taxonomic units (OTUs) when considering SAGs with a predicted completeness of <20% (47). Regardless of completeness, fuzzy boundaries have been suggested and should be considered when using ANI interpretations (48).

### Carbon metabolism.

**FIG 3:**
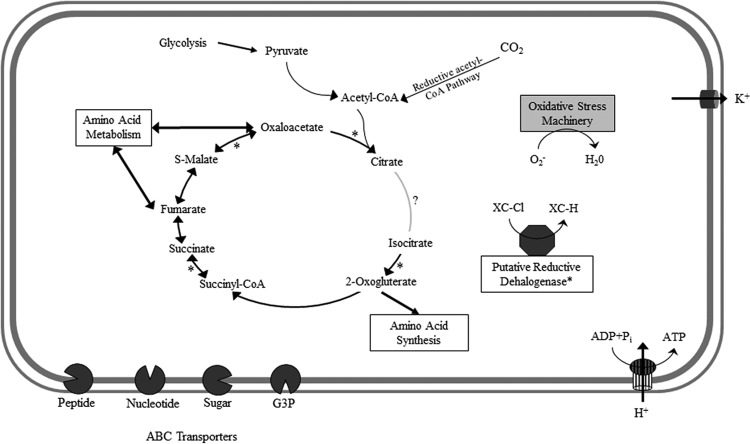
Schematic representing the composite metabolic and transport proteins hypothesized from genome analysis of seven Anaerolineales SAGs. The genes marked with asterisks were identified in only one of the Anaerolineales SAGs.

Predicted central carbon metabolic pathways are illustrated in Fig. 3 as a composite of the seven Anaerolineales SAGs. In contrast to the photosynthetic Chloroflexi, such as Chloroflexus aurantiacus, no genes for the 3-hydroxypropionate cycle were identified (6). At least one gene for the glycolysis/gluconeogenesis pathway, the tricarboxylic acid (TCA) cycle, or the Wood-Ljungdahl pathway is represented in eight of the SAGs (see Tables S2 to S4 in the supplemental material). The single exception is De-I04, which lacks any genes for glycolysis, the TCA cycle, and the Wood-Ljungdahl pathway; however, this genome is very incomplete, with only a predicted 4.5% recovery. Therefore, it is likely that, while a number of genes encoding enzymes catalyzing other central metabolic reactions were recovered, genes for central carbon metabolism were not sequenced rather than being absent from the genome. Comparisons with other strains suggest the complete pathways may be present; however, they were not sequenced from our nine Chloroflexi SAGs.

Metabolic products of glycolysis/gluconeogenesis can either be converted into cellular components or fermented into metabolic waste products. Ethanol is one such waste product, and an alcohol dehydrogenase was identified in six of the SAGs (An-E09, An-B04, An-L07, An-J10, An-B16, and An-B22). This enzyme acts on primary or secondary alcohols to produce a corresponding aldehyde and NADH. Chloroflexi across multiple classes contain annotated alcohol dehydrogenases, including the deep-sea strain DscP2 (16). If alcohols are present within the surrounding environment, they could passively diffuse across the membrane and could be used in central carbon metabolic processes.

In addition to using passive transport, a number of organic carbon transporters were also found. Amino acid, dipeptide, and oligopeptide ABC transporters were identified in the Thermoflexales SAG (Th-L07) and six of the Anaerolineales SAGs (An-L07, An-K11, An-B04, An-J10, An-B16, and An-B22). Xylose, maltose, and multiple-sugar transporters were found in De-I04, An-L07, An-B16, and An-J10. Nucleoside- and deoxyribose-specific transporters were identified in An-K11, An-J10, An-B16, and An-B22. Strains An-L07 and An-K11 encode a glycerol-3-phosphate (G3P) ABC transporter, suggesting exogenous G3P can be utilized in lipid synthesis or glycolysis. ABC transporters are not unique to these Chloroflexi SAGs, as A. thermophila UNI-1 encodes xylose, amino acid, ribose, and carbohydrate ABC transporters. Uptake of these fixed carbon molecules could supply precursors for other anabolic pathways or energy in catabolic pathways, depending on cellular demand. A. thermophila UNI-1 grows by fermentation of sugars at neutral pH (37). These Chloroflexi could be utilizing dissolved amino acids, which have been identified elsewhere in an oceanic crustal aquifer (49).

Besides using exogenous carbon sources, these SAGs encode a number of enzymes in the Wood-Ljungdahl pathway of CO_2_ fixation. A formyltetrahydrofolate synthase, methylenetetrahydrofolate cyclohydrolase, 5,10-methylenetetrahydrofolate dehydrogenase, 5,10-methylenetetrahydrofolate reductase, carbon monoxide dehydrogenase/acetyl-CoA synthase (CODH/ACS), carbon-monoxide dehydrogenase catalytic subunit, and formate dehydrogenase were found in all seven of the Anaerolineales SAGs. A recent metagenomic reconstruction of a putative aerobic Anaerolinea from aquifer sediments adjacent to the Colorado River lacked genes for the Wood-Ljungdahl pathway, pyruvate ferredoxin oxidoreductase, and hydrogenases, which is in contrast to our seven Anaerolineales SAGs (50). Our Dehalococcoidales and Thermoflexales SAGs (De-I04 and Th-L07, respectively) also lack these genes, though in our case, it is likely the genes are missing in the assembly rather than absent from the genome, since the sequenced isolates closest to these SAGs, Dehalogenimonas lykanthroporepellens and T. hugenholtzii JAD2, encode enzymes for the Wood-Ljungdahl pathway (51). Furthermore, an incomplete Wood-Ljungdahl pathway does not prevent the enzymes from being used in other anabolic processes (52). At least one subunit for the pyruvate ferredoxin oxidoreductase was found in four of the SAGs (An-K11, An-B04, An-B16, and An-J10). The protein can provide a link between the Wood-Ljungdahl pathway and other anabolic pathways (53).

In three of the Anaerolineales SAGS, An-B16, An-B22, and An-K11, the formate dehydrogenases were annotated to contain selenocysteine in the active site rather than cysteine. Selenocysteine-specific translation factors were identified in An-B04 and An-B22, but not in An-B16. Interestingly, the encoded formate dehydrogenase of An-J10 is not predicted to contain a selenocysteine, although a selenocysteine-specific translation factor was identified. Like An-J10, the formate dehydrogenases of A. thermophila UNI-1 does not encode a selenocysteine in its active site. Many anaerobic dehydrogenases are annotated to contain selenocysteine in the order Dehalococcoidales; however, there was no evidence of enzymes requiring selenocysteine in the Dehalococcoidales SAG, i.e., De-I04. A. thermophila UNI-1 does not have any dehydrogenases annotated to contain selenocysteine, but it does encode a selenocysteine elongation factor. Selenocysteine-containing formate dehydrogenases are found in many obligate anaerobes and facultative aerobes, suggesting importance under low-oxygen conditions, such as the deep-subsurface biosphere (54). At site C0017, the nitrate concentration at the depth of our sample was less than 2 μM, suggesting an anaerobic environment where oxygen was presumably consumed by microbial respiration within the uppermost layer (12). Genes suggesting selenocysteine usage were found at both sites C0015 and C0017.

### Reductases and hydrogenases.

Genes encoding subunits resembling those of the CoB-CoM heterodisulfide reductase (*hdrABCD*) were identified in five of the Anaerolineales SAGs (An-K11, An-B04, An-J10, An-B16, and An-B22) and were organized in operons. While the enzyme has not been identified in A. thermophila UNI-1, genes for the *hdrABCD* enzymes have been identified in methanogenic archaea and a number of strictly anaerobic bacteria, including the sequenced isolates D. lykanthroporepellens BL-DC-9 and T. hugenholtzii JAD2 (51, 55). Most likely due to low genome coverage, these genes were not identified in the Thermoflexales and Dehalococcoidales SAGs (Th-L07 and De-I04, respectively). The presence of *hrdABCD* in our SAGs supports the hypothesis that these enzymes are responsible for the transfer of reducing equivalents to and from ferredoxins or NADH (17). Conservation across the multiple Chloroflexi lineages suggests that these redox-active enzymes play an integral role in cellular function.

Across all nine of our Chloroflexi SAGs, a number of hydrogenase and hydrogenase accessory proteins were identified. The genes from the hydrogenase maturation apparatus, *hypABCDEF* (56), were identified in An-B16, An-L07, and De-I04. These genes are required for the coordination of the NiFe cofactor and assembly of mature hydrogenase. However, energy-conserving hydrogenase (*ech*), cytoplasmic *hymABC* hydrogenase, and the uptake hydrogenase (*hup*) genes were not specifically identified in any of these nine Chloroflexi SAGs.

### Synthesis and uptake of cofactors.

Hydrogenases and other redox-active enzymes require specific cofactors. Respiratory nitrate reductases, dimethyl sulfoxide reductases, and some carbon monoxide dehydrogenases are all molybdopterin oxidoreductases and therefore require the molybdenum cofactor for activity. The molybdenum cofactor biosynthesis complex *moaABCDE* was represented by one or more genes in six Anaerolineales SAGs (An-E09, An-B04, An-L07, An-J10, An-B16, and An-B22). At least one gene encoding enzymes for molybdopterin cofactor biosynthesis (*moeAB* and *mobAB*) was identified in five of the nine SAGs (De-I04, An-K11, An-B04, An-J10, and An-B16). In addition, molybdenum is required for xanthine dehydrogenase, an enzyme in purine metabolism, which was also identified in An-B04, An-L07, An-J10, and An-B16. Molybdopterin cofactors can coordinate the localization of either molybdenum or tungsten (57). In An-B22, a tungstate ABC transporter was identified, suggesting the organism uses tungsten rather than molybdenum. Both An-J10 and An-B22 contain a vitamin B_12_ ABC transporter.

### Environmental adaptations.

In the Dehalococcoidia SAG DEH-J10, genes for environmental adaptations, such as osmoprotectants (trehalose and alpha-mannosylglycerate synthases) and oxygen protection, were identified (17). A trehalose synthase gene was identified in An-B22, An-L07, and An-B16. Genes for superoxide dismutase and catalase were identified in An-B22, and only the gene for catalase was identified in Th-L07. These gene products could be used to cope with an oxygen influx introduced during ambient seawater mixing with interstitial hydrothermal fluids, as is likely in the more porous sediment layers.

### Cell wall formation.

Terrestrial strains of Chloroflexi lack genes for peptidoglycan synthesis, and D. mccartyi strains contain a proteinaceous surface layer, i.e., an S-layer (18). The enzyme peptidoglycan glycosyltransferase is responsible for joining *N*-acetylglucosamine to *N*-acetylmuramic acid and is essential for peptidoglycan biosynthesis. The gene was identified in SAGs An-B22, An-B04, and An-K11. It is possible that the annotated peptidoglycan glycosyltransferase genes are actually responsible for glycosylation of the S-layer proteins, as suggested for DEH-J10 (17). It has also been suggested that this single cell wall genotype and phenotype is preserved across the phylum Chloroflexi (58).

### Reductive dehalogenase.

**FIG 4:**
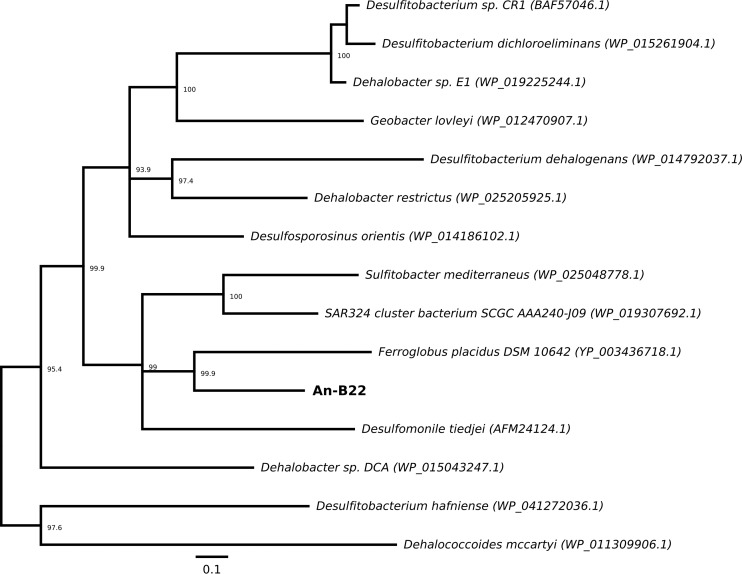
Unrooted Bayesian tree of reductive dehalogenase homologous (RdhA) sequences, including the one identified in Anaerolineales SAG An-B22. The numbers at the nodes represent percent consensus support. The scale bar represents 1 amino acid substitution per 10 positions.

A reductive-dehalogenase gene (*rdhA*) has been directly linked to a subsurface Chloroflexi outside the class Dehalococcoidia. Unlike other SAGs within the Chloroflexi, An-B22 (the largest genome recovered) (Table 1) has an annotated trichloroethene-reductive-dehalogenase gene (*tceA*); however, this annotation must be interpreted cautiously, as RdhA proteins are as yet far from being well characterized. The gene is most closely related to a reductive-dehalogenase gene of the archaeon Ferroglobus placidus DSM 10642 (Fig. 4). Very few reductive dehalogenases have a known function, and this *rdhA* gene is more similar to genes found among known dehalogenators of the Clostridia, Deltaproteobacteria, and Alphaproteobacteria than the Dehalococcoidia (59). Like genes for other reductive-dehalogenase proteins, this *rdhA* gene (from An-B22) encodes binding motifs for an FeS cluster (see Fig. S2 in the supplemental material). In addition, An-B22 encodes a vitamin B_12_ ABC transporter, and the associated corrinoid is a known cofactor of reductive dehalogenases (60).

The reductive dehalogenases of Dehalococcoides are encoded in an operon of *rdhAB* genes. RdhA is the functional dehalogenase, and RdhB is thought to be its associated membrane-bound anchor protein (60). Transcriptional regulators are often encoded adjacent to the *rdhAB* operon, and the operon is often on genomic islands that are horizontally acquired and integrated at the single-copy transfer-messenger RNA (tmRNA) gene, *ssrA* (61). None of these conserved characteristics regarding the reductive dehalogenases of D. mccartyi were found in An-B22 or F. placidus (62). Furthermore, F. placidus has not been shown to conserve energy via reductive dehalogenation (63). Reductive dehalogenases are exported through the membrane fully folded via the TatABC export apparatus (60). A *tatA* gene was identified in An-B22; however, no twin-arginine signal was identified in this *rdhA* gene or in that of F. placidus (62).

Widespread and diverse *rdhA* genes have been detected across the Pacific Ocean in subsurface sediment cores, and microcosms have shown dehalogenation activity (20, 21). Identification of an *rdhA* gene in An-B22 provides phylogenetic linkage to an organism within the phylum Chloroflexi but outside the order Dehalococcoidales. Whether or not this Rdh is involved with organohalide respiration or, more simply, dehalogenation of organic compounds enhancing heterotrophic growth, or perhaps a mixotrophic combination depending upon ephemeral conditions (e.g., oxygen levels), these results, along with previous research (20, 21), support the hypothesis that reductive dehalogenation is an important biogeochemical process within the oceanic deep-subsurface biosphere.

### Conclusions.

This study used single-cell genomics to examine an active subsurface hydrothermal system and to expand the known metabolic functions of uncultured organisms within the phylum Chloroflexi. Photosynthetic Chloroflexi (e.g., C. aurantiacus) can fix carbon through the 3-hydroxypropionate pathway (6), whereas other Chloroflexi isolates (e.g., D. lykanthroporepellens and A. thermophila) use exogenous sources of carbon (35, 51). Although the two sites from the Iheya North hydrothermal field demonstrated hydrodynamic differences, this was not reflected in the SAGs we analyzed. From this study, it remains unclear if these subsurface strains are strictly heterotrophic or autotrophic. Hydrothermal vents can circulate dissolved organic carbon, in addition to inorganic chemicals, into the subsurface biosphere (49). Due to the number of ABC transporters identified across all nine SAGs, it is likely these organisms are living heterotrophically. This hypothesis is further supported by A. thermophila, which also grows heterotrophically by fermentation and contains a number of shared transporters and carbon utilization genes.

These SAGs yield compelling insights to central carbon metabolism and the potential for reductive dehalogenation in an enigmatic group of subsurface microbes. In contrast to previous Chloroflexi SAGs, the genomes were recovered from hydrothermally driven habitats and therefore provide an extended window into the metabolic potential of the deep-subsurface biosphere. Even though the SAGs examined here vary in genome completeness, collectively they have enabled insights into the metabolic potential of subsurface Chloroflexi, providing useful functional gene information with special emphasis on the Anaerolineales.

## Supplementary Material

Supplemental material
