# Supplementary material for: Comparative Single-Cell Genomics of Chloroflexi from the Okinawa Trough Deep-Subsurface Biosphere
Source: Appl Environ Microbiol. 2016 May 2;82(10):3000–8. doi: 10.1128/AEM.00624-16 (PMC4959059; doi:10.1128/AEM.00624-16)
Supplement: Supplemental material [file supp_82_10_3000__index.html]

Comparative Single-Cell Genomics of Chloroflexi from the Okinawa Trough Deep-Subsurface Biosphere — Supplemental material 

# Comparative Single-Cell Genomics of Chloroflexi from the Okinawa Trough Deep-Subsurface Biosphere

## Supplemental material

- Supplemental file 1 -

  Phylogeny and metabolism of select organisms in the phylum *Chloroflexi* (Table S1); one-carbon gene pools (Table S2); glycolysis genes (Table S3); TCA cycle genes (Table S4); estimated genome size based percent genome recovery as determined by tRNAs of nearest neighbor (Table S5); pairwise comparisons of *Chloroflexi* genomes ANI vs. SSU percent identity (Fig. S1); N-terminal amino acid alignment of RdhA sequences (Fig. S2).

  PDF, 359K
