# Supplementary material for: Comparative Single-Cell Genomics of Chloroflexi from the Okinawa Trough Deep-Subsurface Biosphere
Source: Appl Environ Microbiol. 2016 May 2;82(10):3000–8. doi: 10.1128/AEM.00624-16 (PMC4959059; doi:10.1128/AEM.00624-16)
Supplement: Supplemental material [file AEM.00624-16_zam999117131so1.pdf]

Supplementary Information for

Comparative single-cell genomics of *Chloroflexi* from the Okinawa Trough deep subsurface biosphere

H. Fullerton and C. L. Moyer*

*To whom correspondence should be addressed: cmoyer@hydro.biol.wwu.edu

This file contains:

Supplementary Tables S1 - S5

Supplementary Figures S1 - S2

Table S1. Phylogeny and metabolism of select organisms in the phylum *Chloroflexi* .

Organism	Order	Metabolic Classification	Accession Number
Anaerolinea thermophila	*Anaerolineales*	Anaerobic Heterotroph	NC_014960
Caldilinea aerophila	*Caldilineales*	Facultative Heterotroph	NC_017079
Chloroflexus aurantiacus	*Chloroflexales*	Anoxygenic Photoautotroph	NC_010175
Dehalogenimonas lykanthroporepellens	*Dehalococcoidales*	Anaerobic Dehalorespirer	GCA_000143165
Nitrolancetus hollandicus	*Sphaerobacterales*	Chemoautotrophic Nitrite Oxidizer	CAGS01000001
Thermomicrobium roseum	*Thermomicrobiales*	Aerobic Chemoheterotroph	CP001275 (chromosome)
			CP001276 (Megaplasmid)

Table S2. One Carbon Pool Genes

			Dehalococcoidales	Thermoflexales	Anaerolineales						
Carbon Utilization Pathway	Enzyme	EC number	AB-790-I04	AB-790-L07	AB-790-K11	AC-715-B04	AB-790-B16	AC-711-E09	AC-711-L07	AC-711-J10	AC-711-B22
One Carbon pool by Folate	Dihydrofolate reductase	1.5.1.3						0115			
One Carbon pool by Folate	Formyltetrahydrofolate dehydrogenase	1.5.1.6									
One Carbon pool by Folate	Thymidylate synthase	2.1.1.45						0116			
One Carbon pool by Folate	Phosphoribosylglycinamide formyltransferase	2.1.2.3/2.1.2.2	0159c						0803		
One Carbon pool by Folate	5-methyltetrahydrofolate--homocysteine methyltransferase	2.1.1.13					0599, 0651c, 0657c, 0998c		0365	0400	0307
One Carbon pool by Folate	Formimidoyltetrahydrofolate cyclodeaminase	4.3.1.4			0064c					0682	
One Carbon pool by Folate	methionyl-tRNA formyltransferase	2.1.2.9	0081c			0409c				0261	
Wood-Ljungdahl	carbon-monoxide dehydrogenase catalytic subunit	1.2.99.2/1.2.7.4			CooS=0059c				Large Chain=0723		
Wood-Ljungdahl	5,10-Methylenetetrahydrofolate reductase (NAD(P))	1.5.1.20				0343, 0347	0309				0375c
Wood-Ljungdahl	CO dehydrogenase/acetyl-CoA synthase, acetyl-CoA synthase subunit	2.3.1.169			0055c						
Wood-Ljungdahl	Formate dehydrogenase, alpha and delta subunit	1.2.1.2			0279		0768c			0063	0854
Wood-Ljungdahl	Formyltetrahydrofolate synthetase	6.3.4.3			0062c						
Wood-Ljungdahl	5,10-methylenetetrahydrofolate dehydrogenase	1.5.1.5			0063c	0351				0311	0370c
Wood-Ljungdahl	cyclohydrolase	3.5.4.9			0063c	0351				0311	0370c

Table S3. Glycolysis Genes

			Dehalococcoidales	Thermoflexales	Anaerolineales						
Carbon Utilization Pathway	Enzyme	EC number	AB-790-I04	AB-790-L07	AB-790-K11	AC-715-B04	AB-790-B16	AC-711-E09	AC-711-L07	AC-711-J10	AC-711-B22
Glycolysis	Phosphoglucomutase	5.4.2.2				0194	0718c				0165c
Glycolysis	glucokinase	2.7.1.2			0102				0287c		
Glycolysis	isomerase/mannose-6-phosphate isomerase, archaeal	5.3.1.9/5.3.1.8		0333						0652c	1150c
Glycolysis	6-phosphofructokinase	2.7.1.11			0068c		0943c				
Glycolysis	fructose-bisphosphatase	3.1.3.11					0505				
Glycolysis	fructose-bisphosphate aldolase	4.1.2.13					0817c				
Glycolysis	Triosephosphate isomerase	5.3.1.1					0095c				
Glycolysis	glyceraldehyde-3-phosphate dehydrogenase	1.2.1.12					0944c				
Glycolysis	glyceraldehyde-3-phosphate dehydrogenase	1.2.1.59									1092
Glycolysis	Phosphoglycerate kinase	2.7.2.3							0775c		
Glycolysis	2,3-bisphosphoglycerate-independent phosphoglycerate mutase, archaeal type	5.4.2.1			0018c		0606c		0541	0831c	1279
Glycolysis	Enolase	4.2.1.11				0143c	0294				
Glycolysis	Pyruvate Kinase	2.7.1.40					0945c				
Glycolysis	Pyruvate dehydrogenase E1 component	1.2.4.1						α=0146c,<br>β=0147c	α=0012c,<br>β=0013c		
Glycolysis	Pyruvate:ferredoxin oxidoreductase	1.2.7.1			α=0317,<br>β=0318	α=0532c,<br>β=0531c,<br>γ=0534c,<br>δ=0533c	β=1197c			α=0200c,<br>β=0199c,<br>γ=0202c,<br>δ=0201c	
Glycolysis	Dihydrolipoamide dehydrogenase	1.8.1.4			0338		0174c		0297c		
Glycolysis	Acetate-CoA ligase	6.2.1.1				0158	0222c			0250c	

Table S4. TCA Cycle Genes

			Dehalococcoidales	Thermoflexales	Anaerolineales						
Carbon Utilization Pathway	Enzyme	EC number	AB-790-I04	AB-790-L07	AB-790-K11	AC-715-B04	AB-790-B16	AC-711-E09	AC-711-L07	AC-711-J10	AC-711-B22
TCA	Citrate Synthase	2.3.3.1						0089			
TCA	Aconitase	4.2.1.3									
TCA	Isocitrate dehydrogenase	1.1.1.42/1.1.1.41		0084							0381c
TCA	2-Oxoacid:acceptor oxidoreductase	1.2.7.3				α=0512c, β=0488c/0513, γ=0514c, δ=0511	α=0609/0610, β=0134c/0611, γ=0612, δ=0608			α=0075/0544c, β=0076, γ=0077, δ=0074	α=1089, β=1090, γ=0588, δ=0587
TCA	Succinyl-CoA synthetase (ADP-forming)	6.2.1.5									α=1198, β=1196
TCA	Fumarate Reductase	1.3.5.4							C=0522c, D=0521c		
TCA	Succinate Dehydrogenase	1.3.5.1							0523c, 0524c,	0756c	1176c
TCA	Fumarase, class I, aerobic	4.2.1.2				0072, 0073	0778, 0780				0317, 0319
TCA	Malate Dehydrogenase	1.1.1.37		0085							0249c
TCA	Pyruvate/oxaloacetate carboxyltransferase	6.4.1.1					0801c				

Table S5. Estimated genome size based percent genome recovery as determined by tRNAs of nearest neighbor.

SAG Name	Nearest Sequenced Neighbor	SSU % ID to Nearest Neighbor	Estimated Genome Recovery (Based on Nearest Neighbor tRNA Count)	Estimated Size of Genome (Based on tRNAs)
AC-711-E09	*Anaerolinea thermophila* UNI-1	82.54%	4%	3,831,175
AB-790-I04	*Dehalogenimonas lykanthroporepellens* BL-DC-9	87.41%	11%	1,768,736
AB-790-L07	*Thermoflexus hugenholtzii JAD2*	85.62%	16%	2,908,579
AB-790-K11	*Anaerolinea thermophila* UNI-1	80.72%	36%	1,371,942
AC-715-B04	*Anaerolinea thermophila* UNI-1	78.50%	8%	7,697,700
AC-711-L07	*Anaerolinea thermophila* UNI-1	84.27%	22%	3,639,645
AC-711-J10	*Anaerolinea thermophila* UNI-1	78.95%	40%	2,527,060
AB-790-B16	*Anaerolinea thermophila* UNI-1	78.56%	66%	1,906,780
AC-711-B22	*Anaerolinea thermophila* UNI-1	78.47%	52%	2,737,423

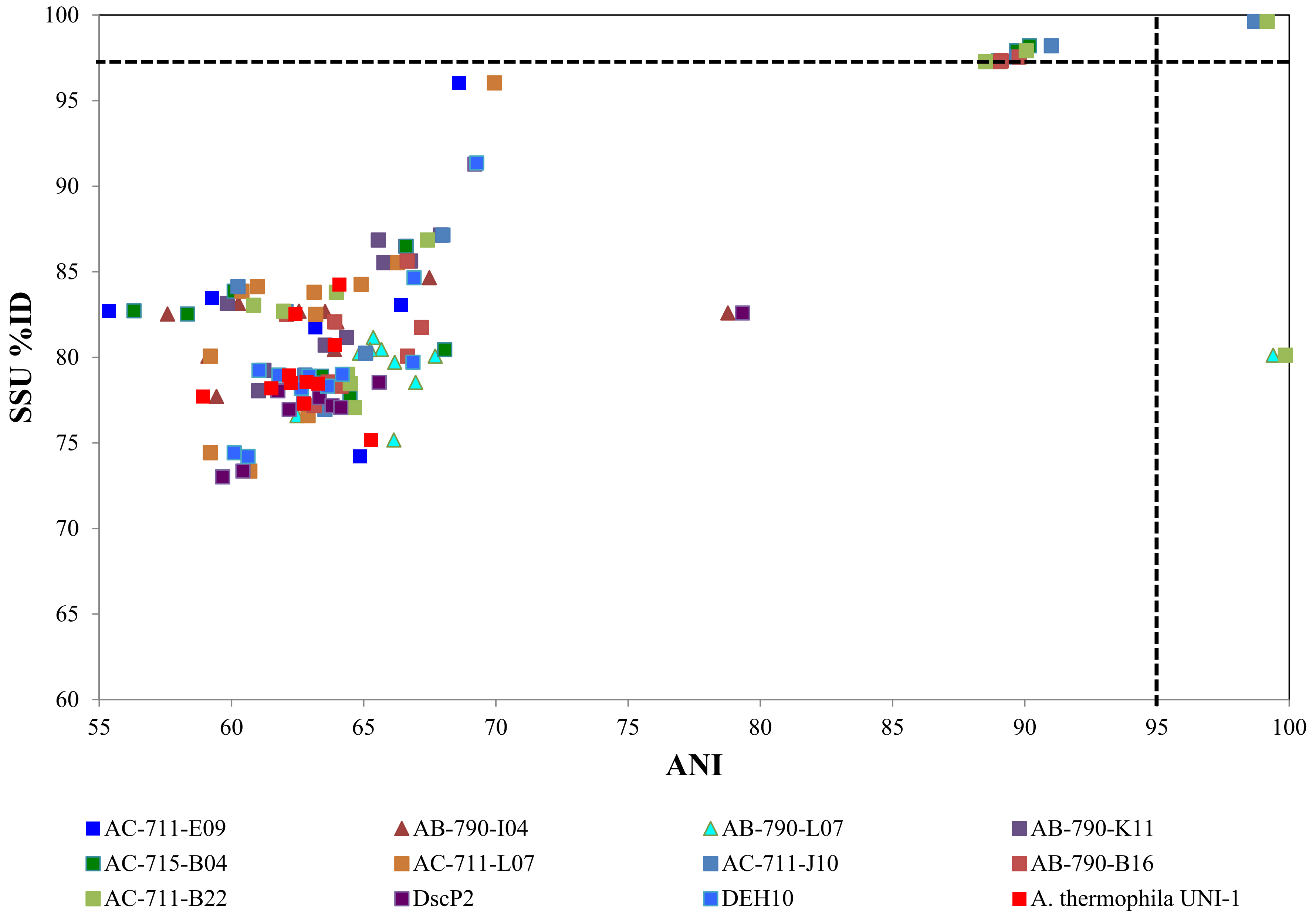

Figure S1. Pairwise comparisons of *Chloroflexi* genomes ANI vs. SSU percent identity. Dashed lines represent species level cutoff values.

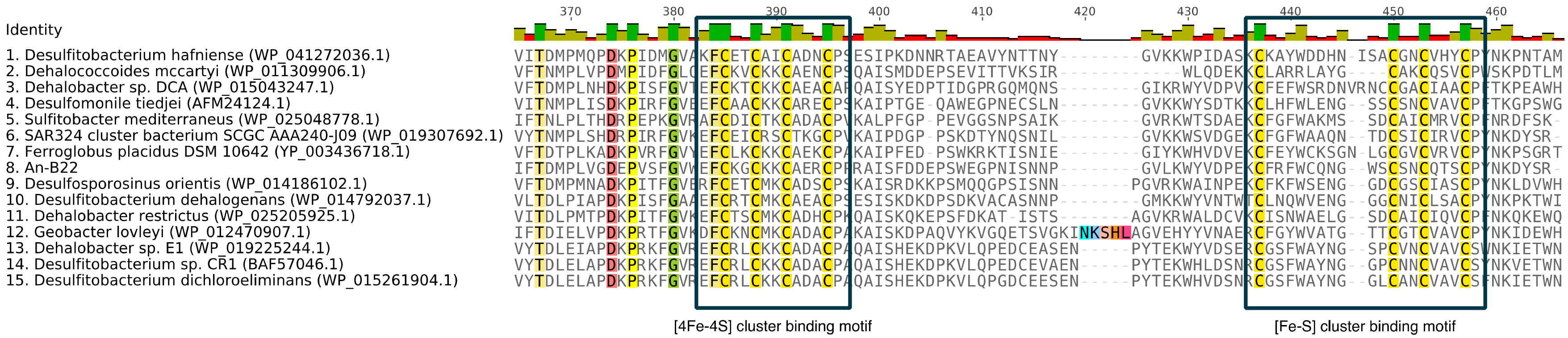

Figure S2. N-terminal amino acid alignment of RdhA sequences, including the one found in SAG An-B22 with boxes highlighting the Fe-S cluster binding motif. This is a subset of the amino acid alignment that was used to create Figure 4.
